# Growth of hopper-shaped CsPbCl_3_ crystals and their exciton polariton emission†

**DOI:** 10.1039/d1ra03977f

**Published:** 2021-07-26

**Authors:** Shiqi Zhao, Tong Guo, Zihao Chu, Yanping Li, Wanjin Xu, Guangzhao Ran

**Affiliations:** State Key Laboratory for Artificial Microstructure and Mesoscopic Physics, School of Physics, Peking University Beijing 100871 China rangz@pku.edu.cn

## Abstract

CsPbCl_3_ is an attractive wide-bandgap perovskite semiconductor. Herein, we have grown hopper-shaped CsPbCl_3_ crystals in a solution droplet dripped on a heated substrate. During the growth, we have observed the impacts of the coffee ring effect and Marangoni flow, which may result in the hopper shape. Their photoluminescence spectra feature double peaks, which are located at 413.9 nm and 422.0 nm, respectively, and the latter increases faster in intensity than the former as the excitation power increases. We believe that the higher-energy peak originates from the excitonic emission and the lower-energy one is from the polaritons' emission, where the polaritons are generated in the exciton–exciton inelastic scattering process. Based on such an explanation, the exciton binding energy of CsPbCl_3_ is estimated to be 76.7 meV in our experiments, consistent with the previous reports.

## Abstract 2

We report hopper-shaped CsPbCl_3_ crystals grown in a solution droplet and the polariton emission from them.

## Graphical abstract

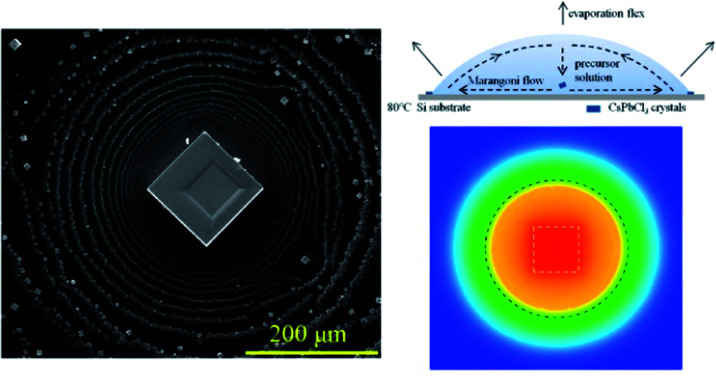

## Introduction

In recent years, halide perovskites have been intensively studied because of their low-cost solution fabrication process and their outstanding optoelectronic properties. Among them, inorganic perovskites have attracted special attention because of their better environmental stability, especially compared to organic perovskite materials.^1–3^ As a kind of deep blue light emitting material, CsPbCl_3_ has been widely studied in lasers^4–6^ and light-emitting devices.^7^ But because of the very low solubility of the precursor,^8^ it is difficult to prepare large sized CsPbCl_3_ crystals in solution. Furthermore, the surfaces of the growing CsPbCl_3_ crystals are usually rough due to the spontaneous nucleation and unstable growth when the degree of supersaturation is too high. In order to obtain CsPbCl_3_ crystals with smooth surfaces, Gui *et al.* adopted a space-confined growth method and obtained CsPbCl_3_ microplatelets with sizes of about 30 μm × 60 μm;^8^ and, by using a similar method, He *et al.* obtained microdisk arrays of CsPbCl_3_ for lasing;^9^ moreover, Hu *et al.* used soft templates of CH_3_(CH_2_)_3_NH_3_I micelles in the solution to grow CsPbCl_3_ crystals.^10^

However, when the degree of supersaturation is finely adjusted at an intermediate degree of the interfacial instability, hopper shapes can form in the surface of the perovskite crystals when grown using a solution method.^11–18^ Chen *et al.*,^11^ Zhang *et al.*^15^ and Li *et al.*^17^ obtained hopper shaped CH_3_NH_3_PbX_3_ (X = Br, I) perovskite crystals, and attributed it to the dissolution of the crystals due to the low concentration of the precursor in the central part. In the work of Hou *et al.*, the local enrichment of methylamine caused the dissolution of the CH_3_NH_3_PbBr_3_ crystal surface.^16^ These hopper-shaped crystals are unique in morphologies and usually have some associated properties and applications.^19^

In this work, we have grown hopper-shaped CsPbCl_3_ crystals for the first time in a solution droplet dripped on a heated substrate and tentatively clarify the growth mechanism. They have double-peaked photoluminescence (PL) spectra, one of which we attributed to the exciton polariton emission in the inelastic exciton–exciton scattering process.

## Experimental section

### Materials

PbCl_2_ (99.99%) and CsCl (99.9%) were purchased from Xi'an Polymer Light Technology Corporation (China), and dimethyl sulphoxide (DMSO, chromatographic grade) from Sigma-Aldrich (USA). The materials were used as received without any further purification.

### Methods

The precursor solution was prepared by mixing CsCl and PbCl_2_ with a 1 : 1 molar ratio in DMSO solvent, and then the 0.05 M CsPbCl_3_ precursor solution was stirred at 70 °C for more than 10 hours until the solution became completely transparent. The Si substrate was washed with acetone, ethanol and deionized water, and then transferred onto a heater and preheated at 80 °C. The filtered precursor solution was drop-cast onto the heated Si substrate and maintained at 80 °C for 30 min to let the solution droplet evaporate completely. Finally, the CsPbCl_3_ crystals were obtained.

### Characterization

Surface morphology images were taken on a high-resolution field emission scanning electron microscope (SEM, FEI Quanta 200F) with an accelerating voltage of 15 kV, also equipped with Energy Dispersive Spectrometer (EDS) and cathodoluminescence (CL). Optical absorption was measured on a UV-VIS-NIR spectrophotometer (Shimadzu UV3600 Plus). PL lifetime was measured on a lifetime and steady state spectrometer (FLS980, Edinburgh, UK). X-ray diffraction (XRD) patterns were measured using X-ray diffraction platform (Model PANalytical X'Pert Pro) with a Cu-Kα radiation source (*λ* = 0.1541874 nm) at 40 kV and 40 mA. The angle accuracy was within ±0.0025°. PL was excited at 355 nm by a pulsed Nd:YAG laser (pulse width 8.0 ns, repetition rate 1.0 kHz).

## Results and discussion

**Fig. 1:**
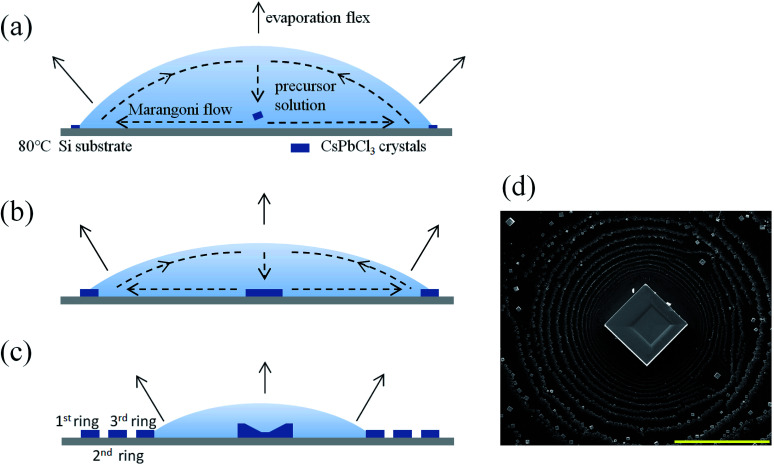
(a–c) Schematic diagram of the growth process for CsPbCl_3_ crystals. (d) SEM image of a CsPbCl_3_ crystal and the coffee rings around it. The scale bar is 200 μm.

Fig. 1(a)–(c) illustrate the growth strategy in a solution phase. As shown in Fig. 1(a), 50 μL of the 0.05 M CsPbCl_3_ precursor solution is dropped onto an 80 °C Si substrate and the solution droplet forms and begins to evaporate. Because of the sharp wedge-shaped boundary, the droplet evaporates heavier and thus supersaturates first in the boundary regime, and then nucleation and crystallization occur first there. Because of the pinning of the contact line, more and more small CsPbCl_3_ crystals appear on the contact line and grow up into a few microns scale, forming one circle eventually, similar to the coffee ring. After that, the evaporating droplet decreases in volume and the contact angle becomes smaller as shown in Fig. 1(b). When the contact angle is smaller than the receding angle of the droplet on the Si substrate, the contact line depines and the droplet shrinks abruptly. When the contact line is pinned again, the above growth process will re-occur at the droplet boundary, and the second ring will form. Finally, a pattern with several concentric “coffee rings” is formed as shown in Fig. 1(c) schematically and as demonstrated by the SEM image in Fig. 1(d). The side length of the resulting cubic crystals in the ring regime ranges from 1 to 5 μm. As contrast, the largest crystal locating at the center of the ring is about 50 μm in size with an aspect ratio about 2, and the location of which is related to Marangoni flow as indicated.^20^ Because the temperature at the vertex of the droplet is lower than other part, the gradient in temperature results in a gradient in surface tension, and finally generates a Marangoni flow in the indicated directions. One CsPbCl_3_ crystal that originally forms at the droplet boundary is transported to the center of the droplet and drops on the substrate and grows up there. Noticeably, there is a remarkable square depression in the middle of the CsPbCl_3_ square crystal. The impacts of the substrate temperature and solution concentration are shown in Fig. S1(a–e) in ESI.† The origin of the depression will be discussed in detail later.

**Fig. 2:**
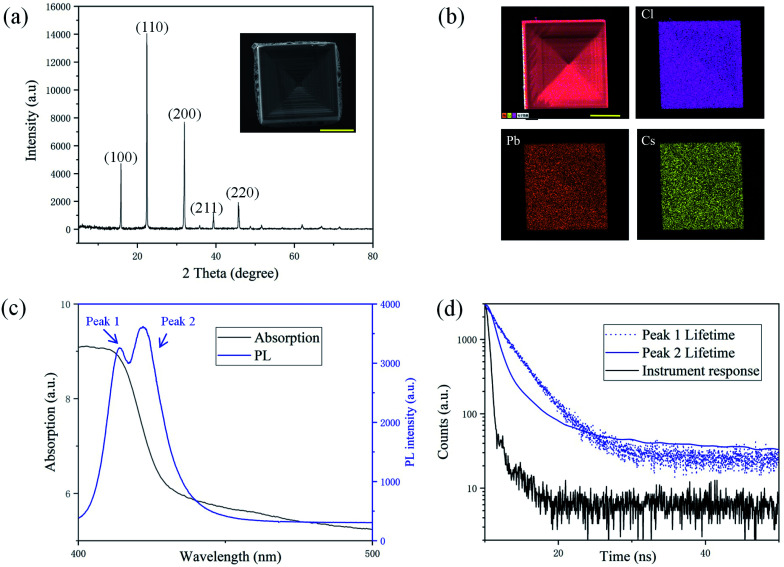
(a) XRD patterns of CsPbCl_3_ crystals. The inset is the SEM image of a typical CsPbCl_3_ crystal. The scale bar is 20 μm. (b) EDS mapping images of the CsPbCl_3_ crystal. The scale bar is 25 μm. (c) The optical absorption spectrum and PL spectrum of the CsPbCl_3_ crystal. (d) PL lifetime spectra of the CsPbCl_3_ crystal and the instrument response.

The SEM image of a typical square crystal with depressions is shown in Fig. 2(a) as an inset, which is grown in the bottom central regime of the droplet and is characteristic of a hopper-shape. The XRD peaks of the crystals shown in Fig. 2(a) at 15.80°, 22.41°, 31.91°, 39.34° and 45.75° can be assigned to (100), (110), (200), (211) and (220) planes of the cubic phase CsPbCl_3_.^21^ The EDS spectra of Cs, Pb and Cl shown in Fig. 2(b) confirm the atomic ratio of Cs, Pb, and Cl to be 17.2 : 20.2 : 62.6, close to the ratio of 1 : 1 : 3 in CsPbCl_3_. The absorption spectrum of the crystal is shown in the black solid line in Fig. 2(c) and the absorption edge is estimated to be at 413.0 nm. As shown in the blue solid line in Fig. 2(c), the typical PL spectrum of the hopper-shaped CsPbCl_3_ sample has two peaks at 413.9 nm (peak 1) and 422.0 nm (peak 2) distinctly. The fitted lifetime of peak 1 is 2.9 ns, and that of peak 2 is 1.4 ns as shown in Fig. 2(d), evidently shorter than the former one. The structure and compositions of the central crystal and those in the rings are compared in Fig. S2(a–c), Table S1 and Fig. S3(a–c) in ESI.† It can be seen from Fig. S3(a–c)† that the CL spectra of the central crystals are all double-peaked, and those of rings are all single-peaked, which shows that the double-peaked CL/PL structure is related to the “hopper-shaped” structure.

**Fig. 3:**
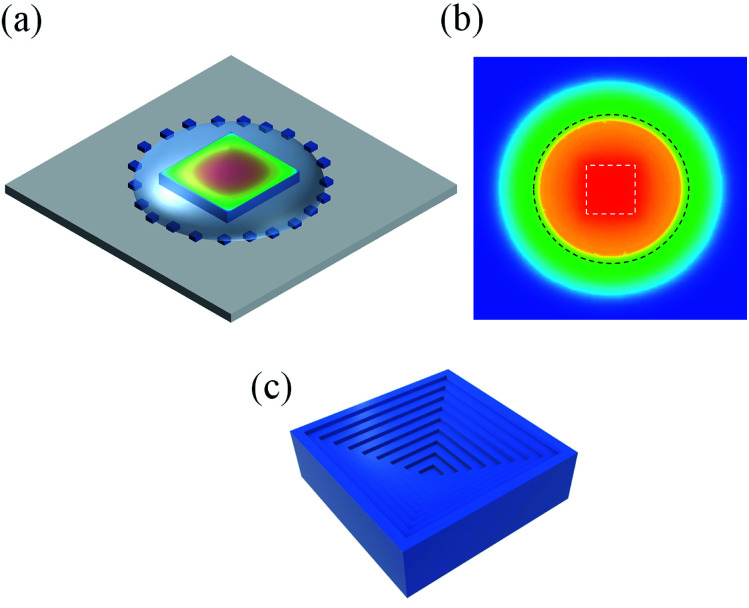
(a) The simulated growth conditions of the hopper-shaped CsPbCl_3_ crystal. The color map calculated by COMSOL shows the temperature distribution on crystal surface at the height of the grown crystals. From the central of the color map to the outer range, the temperature decrease gradually. The temperature difference is nearly 1 °C. (b) The temperature distribution calculated by COMSOL in the droplet section and around the droplet at the top surface of the grown crystals. The white dotted line indicates the position of the crystal, and the black dotted line shows the intersecting line of this plane and the droplet. From the central of the color map to the outer range, the temperature decreases about 4 °C. (c) The schematic hopper-shaped CsPbCl_3_ crystal grown in the conditions shown in (a).

Next, we focus on the growth mechanism of the hopper-shaped CsPbCl_3_ crystals. The habit of crystal growth is determined by its internal structure and the external growth conditions, such as concentration distributions and temperature. The first factor for the hopper shape is the non-uniform evaporate rate. The precursor solution droplet has a sharp-edged boundary, and thus has a higher evaporation rate there, which can be verified by the diffusion equation.^22^ As a result, the solution concentration is higher near the boundary, thus the concentration at the edges of the growing crystal is higher than that in the central regime of the crystal, and the growth is faster at the crystal edges. The second factor is the temperature distribution. The precursor solution droplet on the surface of the crystal acts as a heat-insulating layer, thus the temperature in the central regime of the surface of the crystal is higher as indicated by the color map in Fig. 3(a), which is calculated by COMSOL. From the central of the color map to the outer range, the temperature decreases gradually, and the temperature difference is nearly 1 °C in the range of 25 μm. And Fig. 3(b) shows the temperature distribution calculated by COMSOL in the droplet section and around the droplet at the height of the top surface of the grown crystals. The white dotted line indicates the position of the crystal, and the black dotted line shows the intersecting circle of this plane and the droplet. From the central of the color map to the outer range, the temperature decreases about 4 °C in the range of 500 μm. Because the solubility of CsPbCl_3_ decreases with the decrease of temperature, the degree of the supersaturation is higher around the square crystal and thus it grows faster there. Eventually, the hopper shape, consisting of a series of depression steps, appears on the CsPbCl_3_ crystal surface as shown in Fig. 3(c). The repeatability of the growth of the hopper shaped crystals is shown in Fig. S5(a–c).†

**Fig. 4:**
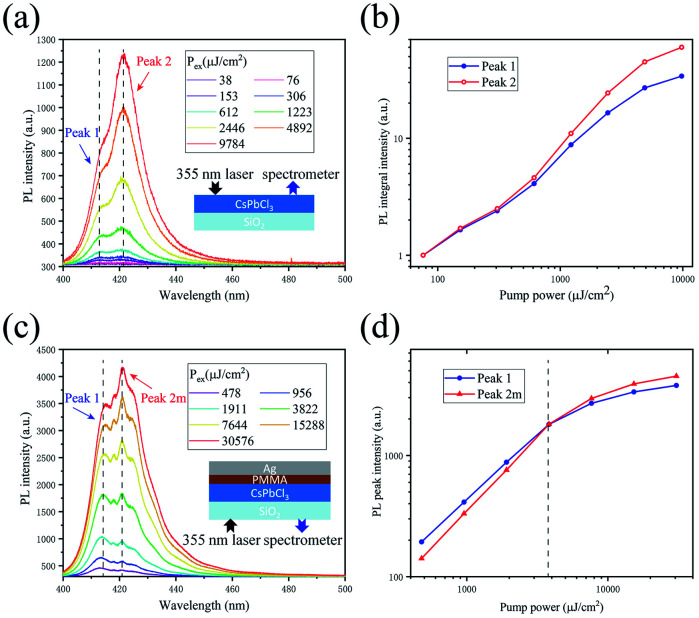
(a) The evolution of PL spectra under different pump fluences. The emitting structure is shown as an inset. (b) The integrated intensities of peak 1 and peak 2 as a function of excitation fluences. (c) The evolution of the PL spectra under different pump fluences. The emitting structure is shown as an inset. (d) The PL peak intensities of peak 1 and peak 2m as a function of excitation fluences.

Now, we study their double-peaked PL spectra. A series of PL spectra under different pump fluences are shown in Fig. 4(a), and the integrated intensities of peak 1 and peak 2 *versus* excitation powers are compared in Fig. 4(b). The peak analyses are shown in Fig. S4(a–d).† We can see that the intensity of peak 2 increases much faster than that of peak 1 with the increase of excitation power, similar to the previous reports by Kunugita *et al.*^23^ and Chen *et al.*,^24^ where the lower-energy PL peak is called polariton-emission. After pumping, the exciton density near the phonon bottleneck regime is so high that they are prone to scattering. After scattering, one exciton is scattered into a higher exciton state with *n* = 2, and another one is scattered into a photon-like polariton state with lower energy and recombines radiatively there, which is so called polariton-emission.^23–28^ In our case, peak 1 originates from *n* = 1 excitons and peak 2 corresponds to polariton-emission, between which the energy difference can be inferred from the transition energy from the *n* = 1 to the *n* = 2 exciton state, equal to 3/4 of exciton binding energy.^23,27^ The energy difference in Fig. 4(a) is 57.5 meV, thus exciton binding energy of the CsPbCl_3_ crystal is estimated to be 76.7 meV, consistent to the result reported previously.^29^ With higher excitation level, more exciton scatterings will suppress the increase of exciton emission (peak 1) and enhance that of peak 2, which is the reason for the faster increase of the peak 2 in intensity. The higher exited states (*n* > 2) of the exciton will relax to lower excited states immediately (∼ps) with the assistance of the phonons at room temperature, thus it is hard to observe the emission from *n* = 3, 4, *etc.* in our experiment. In order to further study the optical properties of the crystal, a layer of 50 nm PMMA is spin-coated on the CsPbCl_3_ crystals grown on glass to prevent exciton quenching when optically excited, then a 200 nm layer of Ag is deposited on the surface by thermal evaporation as a reflector, as shown in Fig. 4(c). In such a microcavity, some weak modes appear, and the mode at 420.9 nm (peak 2m) grows fastest, which exceeds peak 1 in intensity at 3822 μJ cm^−2^, as shown in Fig. 4(d); however, in the further higher power regime, its increasing rate slows down. Finally, we have examined the PL stability of the crystals in Fig. S6.†

## Conclusions

In summary, we have grown hopper-shaped CsPbCl_3_ crystals in a solution droplet, and investigated their growth mechanism by comparing the temperature distribution and evaporation rate of the solution droplet. The crystals have double-peaked PL spectra and the peak with lower energy is believed to originate from the exciton polariton emission, and its PL intensity increases faster than that of the other peak with the increase of excitation power.

## Author contributions

Shiqi Zhao: conceptualization, data curation, formal analysis, software, investigation, validation, visualization, writing – original draft, writing – review & editing. Tong Guo: validation, visualization. Zihao Chu: investigation, validation. Yanping Li: methodology, resources. Wanjin Xu: methodology, resources. Guangzhao Ran: supervision, methodology, project administration, resources, writing – review & editing.

## Conflicts of interest

There are no conflicts to declare.

## Supplementary Material

RA-011-D1RA03977F-s001
