# Supplementary material for: Growth of hopper-shaped CsPbCl3 crystals and their exciton polariton emission
Source: RSC Adv. 2021 Jul 26;11(41):25653–7. doi: 10.1039/d1ra03977f (PMC9037009; doi:10.1039/d1ra03977f)
Supplement: RA-011-D1RA03977F-s001 [file RA-011-D1RA03977F-s001.pdf]

Electronic Supplementary Material (ESI) for RSC Advances.
This journal is © The Royal Society of Chemistry 2021

Supporting Information

Growth of hopper-shaped $CsPbCl_3$ crystals and the exciton polariton emission

*Shiqi Zhao, Tong Guo, Zihao Chu, Yanping Li, Wanjin Xu and Guangzhao Ran**

State Key Laboratory for Artificial Microstructure and Mesoscopic Physics, School of Physics, Peking University, Beijing 100871, China.

*Corresponding Author: rangz@pku.edu.cn

Section 1. The preparation of $CsPbCl_3$ under different solution concentrations and substrate temperatures.

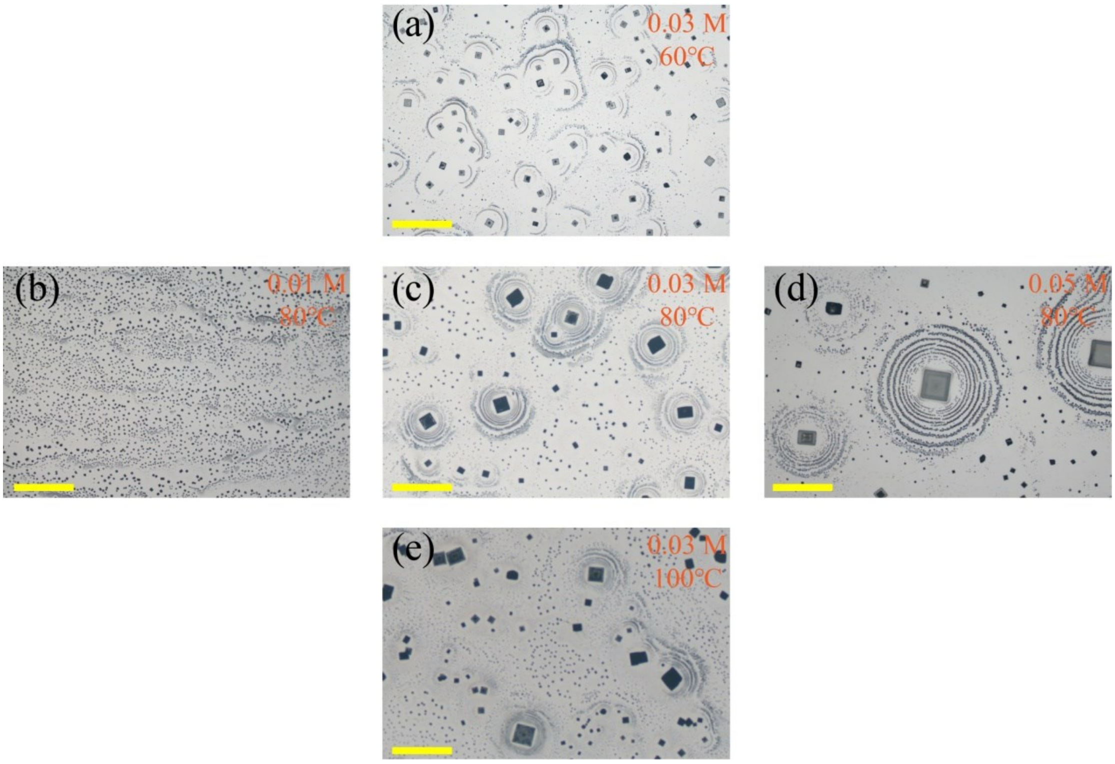

Fig. S1 Typical optical microscope images of the central $CsPbCl_3$ crystals and the ring patterns grown on the Si substrate under different substrate temperature and solution concentration as indicated in each image. All scale bars are 200 μm. (a) 60 ^{o}C and 0.03 M; (b) 80 ^{o}C and 0.01 M; (c) 80 ^{o}C and 0.03 M; (d) 80 ^{o}C and 0.05 M; (e) 100 ^{o}C and 0.03 M. In the row direction, the solution concentration varies; in the column direction, the substrate temperature varies.

For the three images in the row, the solution concentration varies from 0.01 M to 0.03 M and 0.05 M and the substrates temperature is kept to be 80 ^{o}C. In the column direction, the substrate temperature varies. Generally, as the solution concentration increases, larger central crystals are more likely to appear. Under the same concentration of 0.03 M, lower substrate temperature of 60 ^{o}C leads to the smaller sizes of crystals and the small ring patterns; higher substrate temperature of 100 ℃ leads to larger crystals but many of the rings disappear or open. So we can approximately control the size of central crystals and the pattern of the rings, but can not accurately.

Section 2. Comparison of the composition and structure between the central crystals and those in the rings by Energy Dispersive Spectrometer (EDS) and cathodoluminescence (CL).

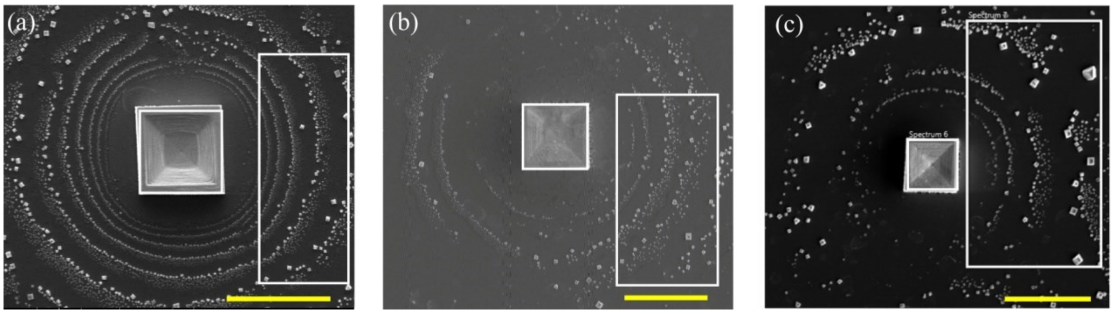

Figs. S2(a-c) Scanning electron microscope (SEM) images of $CsPbCl_3$ crystal. All scale bars are 50 μm. The marked square and rectangle frames stand for the collecting areas of the EDS signals. Due to the weak signal in the ring regions, their collecting areas are larger.

Table S1 EDS data collected from the central crystals and those in the rings marked in Figs. S2(a-c) and the difference between them

	Sample	Cs (%)	Pb (%)	Cl (%)
Central	(a)	21.02	20.65	58.33
	(b)	20.24	20.76	59.00
	(c)	20.41	20.53	59.06
	average	20.56	20.65	58.80
Rings	(a)	19.42	18.71	61.87
	(b)	20.1	20.74	59.16
	(c)	19.61	20.81	59.58
	average	19.71	20.09	60.20
Difference	average	0.85	0.56	-1.41

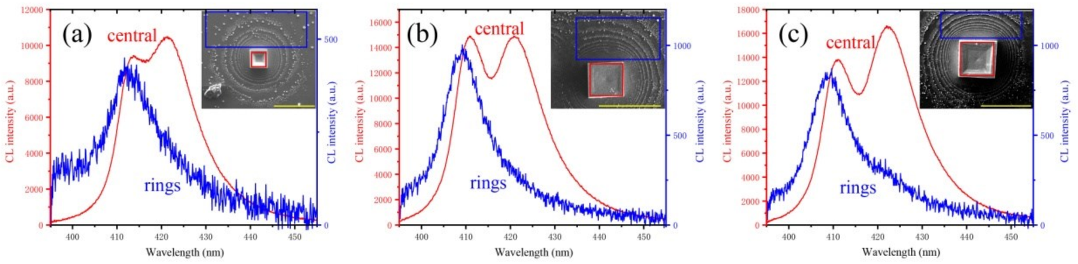

Figs. S3(a-c) CL spectra for three typical $CsPbCl_3$ crystals and the rings around them, and the insets are their SEM images. All scale bars are 100 μm. The marked red square and blue rectangle frames stand for the collecting areas of the CL signals. Due to the weak signals in the ring regions, their collecting areas are evidently larger. The red and blue curves show the intensities of CL in the central crystal and the rings marked in red and blue frames, and correspond to right and left axes, respectively.

From EDS data listed in Table S1, the difference between central crystals and those in the rings is so small that we can believe they both have $CsPbCl_3$ crystal structure and compositions; however, as shown in Fig S3, the CL spectra of the central crystals are all double-peaked, and those of rings are all single-peaked, which shows that the double-peaked CL/ PL structure is related to the "hopper-shaped" structure.

Section 3. PL spectra fitting results at three different fluences

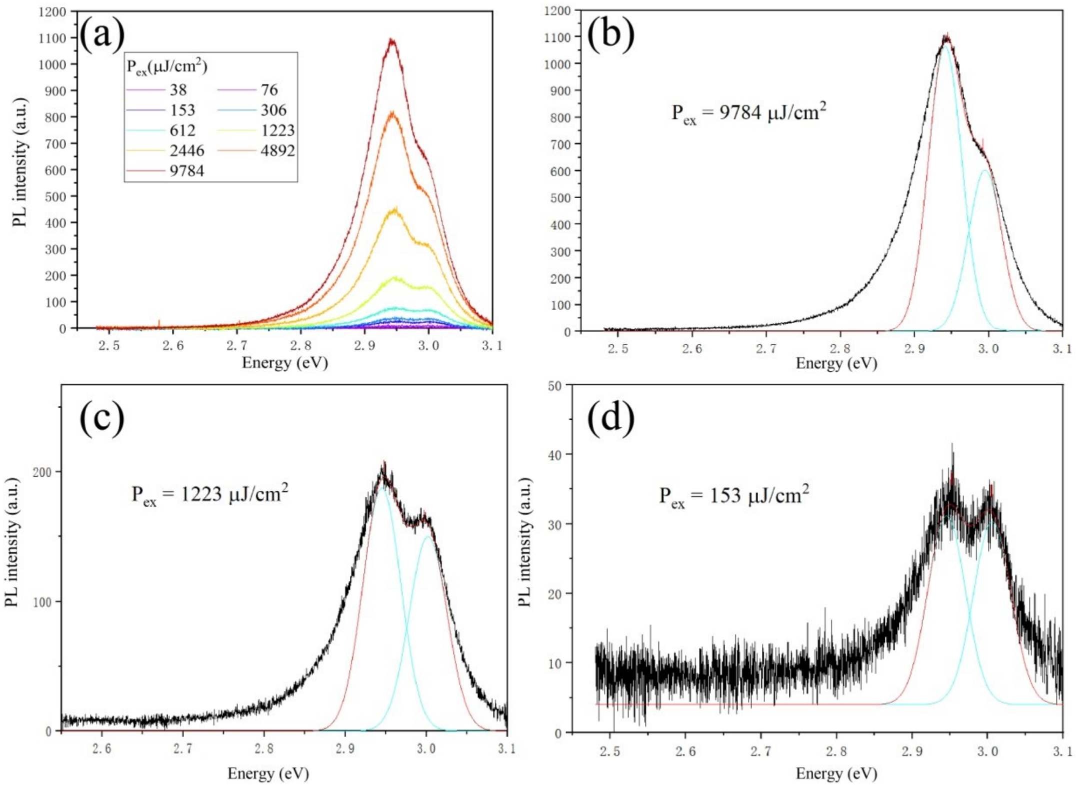

Fig. S4 (a) The evolution of PL spectra under different pump fluences with photon energy as unit of x-axis. PL spectra fitting results at (b) 9784 μJ/cm^2, (c) 1223 μJ/cm^2, and (d) 153 μJ/cm^2. The black line is experimental data. The two blue lines fit the two peaks respectively. The red line is the sum of the fitting results of the two peaks.

There is still a gap between the fitting results and the experimental data, the reason of which may be that our PL spectra had not been calibrated according to the system response function.

Section 4. Repeatability and productivity of the growth of the hopper shaped crystals.

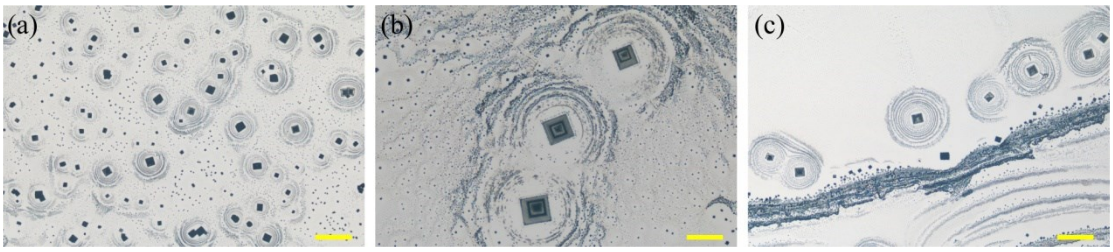

Fig. S5 $CsPbCl_3$ crystals prepared three times with 0.05 M precursor solution. Each time-lag between (a) and (b) and between (b) and (c) is 5 months. All scale bars are 100 μm.

The microscope images in Figs. S5(a-c) are obtained in three times. There are all typical hopper-shaped crystals surrounded by the ring pattern on the Si substrates. It shows that the repeatability of the growth of hopper-shaped $CsPbCl_3$ crystals is good, but the shape and size of the crystals can not be controlled exactly due to the complexity of the solution synthesis.

Section 5. PL stability.

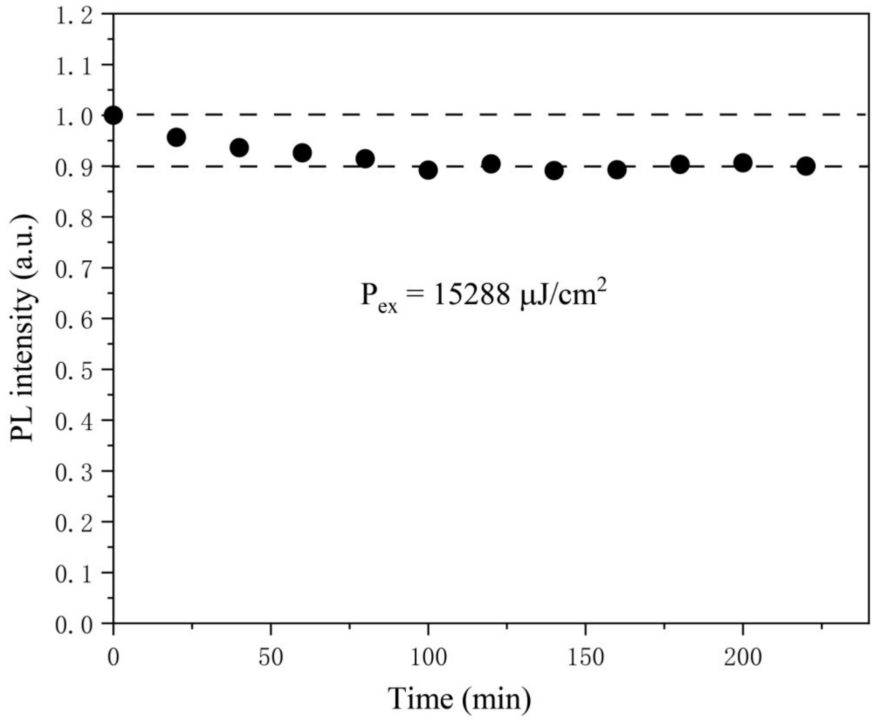

Fig. S6 The PL stability test under a high humidity of 60% and room temperature by an intense constant UV laser source (355nm, pulse width 8.0 ns, repetition rate 1.0 kHz).

The PL stability test under a humidity of 60% and room temperature by an intense constant UV laser source is shown in Fig. S6. When the pump fluence is 15288 μJ/cm^2, PL intensity decreases about 10% in 220 minutes.
